# Lycopene: Hepatoprotective and Antioxidant Effects toward Bisphenol A-Induced Toxicity in Female Wistar Rats

**DOI:** 10.1155/2018/5167524

**Published:** 2018-07-26

**Authors:** Haidy G. Abdel-Rahman, Heba M. A. Abdelrazek, Dalia W. Zeidan, Rasha M. Mohamed, Aaser M. Abdelazim

**Affiliations:** ^1^Department of Clinical Pathology, Faculty of Veterinary Medicine, Suez Canal University, Ismailia, Egypt; ^2^Department of Physiology, Faculty of Veterinary Medicine, Suez Canal University, Ismailia, Egypt; ^3^Department of Home and Economics, Nutrition and Food Science Branch, Faculty of Education, Suez Canal University, Ismailia, Egypt; ^4^Department of Forensic Medicine & Clinical Toxicology, Faculty of Medicine, Suez Canal University, Ismailia, Egypt; ^5^Department of Biochemistry, Faculty of Veterinary Medicine, Zagazig University, Zagazig, Egypt; ^6^Department of Basic Medical Sciences, College of Applied Medical Sciences, University of Bisha, Bisha, Saudi Arabia

## Abstract

Bisphenol A (BPA)—an endocrine disruptor xenoestrogen—is widely spread in the environment. Lycopene (LYC) is an antioxidant phytochemical carotenoid. The hereby study was designed to verify the deleterious effect of BPA on cyclic female rats' hepatic tissue as well as evaluation of the effect of LYC toward BPA hepatic perturbation. Twenty-eight female Wistar rats were allocated equally into four groups: control group, LYC group (10 mg/kg B.wt), BPA group (10 mg/kg B.wt), and BPA + LYC group (the same doses as former groups). The treatments were given daily via gavage to the rats for 30 days. The rats in BPA displayed high activities of serum liver enzymes with low levels of total proteins (TP) and albumin. Moreover, BPA induced hepatic oxidative stress via depletion of antioxidant enzymes concomitant with augmentation of lipid peroxidation, increased comet tail DNA %, and overexpression of caspase-3. Meanwhile, LYC administration reduced the cytotoxic effects of BPA on hepatic tissue, through improving the liver function biomarkers and oxidant-antioxidant state as well as DNA damage around the control values. These findings were confirmed by hepatic histopathological examination. Finally, LYC credited to have a noticeable protective effect versus BPA provoked oxidative injury and apoptosis of the liver tissue.

## 1. Introduction

Liver is the major organ in the body responsible for detoxification and metabolism, resulting in the production of free radicals, which are very reactive and unstable [1]. These products are eliminated by antioxidants that are naturally present in the tissue [2]. The imbalance between the free radical production and elimination leads to oxidative stress which can cause hepatic damage [3].

Bisphenol A (BPA)—an endocrine disruptor—is a monomer used in polycarbonate plastic industry and epoxy resins that lines cans of preserved food and beverages [4]. It can immigrate into food or water on heating [5]. Due to the wide spreading of the usage of BPA in manufacture, both animals and human are daily exposed to BPA hazardous effects [6]. BPA is metabolized primarily in the liver [7]. It has been reported that BPA can cause hepatic [8], renal [9], cerebral [10], and other organs damage by producing reactive oxygen species (ROS) [11]. Moreover, BPA lead to lipid peroxidation of the hepatic tissue by diminishing the endogenous antioxidant defense mechanism in male rats [12].

Antioxidants are present naturally in the living cells as superoxide dismutase, catalase, glutathione reductase, and glutathione oxidase [13] or are available in food such as carotenoids, polyphenols, and vitamins C and E [14]. Therefore, there is a dietary trend recommending increased intake of plant foods rich in antioxidants as an attempt to protect from diseases [15].

An interest has been aroused in utilizing natural products isolated from plants against chemical compounds generating tissue damage [16]. Lycopene (LYC) is a natural antioxidant and free radical scavenger lipophilic carotenoid present in food especially tomatoes giving it the red color [17]. LYC supplementation had been tested for its ameliorative effect against the harmful oxidative injury of tissues caused by environmental toxicants [18, 19]. Additionally, it can retrieve the peroxyl radicals, thus restraining lipid peroxidation pathway [20]. In view of the aforesaid literatures, the exposure of cyclic female rats to BPA has not been fully elucidated. Also, there has been no model exploring the effect of lycopene on BPA exposure; thus, the current study aimed at examining whether LYC has a potential protective action against BPA-induced hepatotoxicity in cyclic female rat model with concern to biochemical, oxidative stress, and antioxidant capacity in the liver tissue as well as histopathological alterations, percent of DNA in comet tail, and hepatic caspase-3 protein contents.

## 2. Materials and Methods

### 2.1. Animals and Experimental Design

Twenty-eight female Wistar rats weighing 94–100 g were bought from the Egyptian Organization for Biological Products and Vaccines, Helwan, Egypt. They were kept two weeks for accommodation prior to the onset of the experiment. Rats were kept in wire-topped cages and housed in a ventilated room under standardized housing conditions of natural light/dark rhythm, temperature 25 ± 2°C, and humidity 48% ± 2. Rats were given ad libitum diet and drinking water. The design of this experiment was approbated by the Research Ethical Committee of Faculty of Veterinary Medicine, Suez Canal University, Egypt. Rats were randomly allotted to four experimental groups, seven rats each. The 1st group received corn oil and considered as a control. The 2nd group was given LYC (NOW FOODS Co., USA) at a dose of 10 mg/kg B.wt [17] daily via gavage. The 3rd group was given BPA (Sigma-Aldrich Co., USA) at 10 mg/kg B.wt [21] daily via gavage. Finally, the 4th group was administrated both BPA and LYC at the same doses of the 2nd and 3rd groups daily via gavage. All treatments continued for 30 days.

### 2.2. Body and Liver Weights

Rats were weighed at the beginning of the experiment and then weighed at the end of the experiment. The body weight gain (B.wt.G) was calculated by subtracting the initial from the final body weight (F.B.wt). The relative liver weight was calculated as follows: absolute liver weight at the end of experiment/F.B.wt × 100.

### 2.3. Serum and Tissue Sampling

At the end of the experiment, female rats at luteal phase of estrous cycle (diestrus) were anesthetized via diethyl ether inhalation. Blood samples were collected from retroorbital venous plexus of the eye into clean plain tubes and left for clot formation, and then, sera were collected and stored at −20°C to evaluate immediate the hepatic function biomarkers and lipid profile.

Thereafter, rats were immolated by cervical dislocation. Liver, from each rat, was immediately enucleated, washed out with buffer saline, blotted by filter paper, and then weighed. A part of the liver from each rat was preserved in 10% neutral formalin for the histopathological and immunohistochemical investigations. The remaining parts were divided into two parts and kept at −80°C. The first part was used for liver tissue homogenate preparation to estimate antioxidant enzymes, lipid peroxidation, and cytochrome P450 reductase (CYPR450) assessments. The second part was used for single-cell suspensions followed by comet assay procedures.

### 2.4. Serum Biochemical Analysis

#### 2.4.1. Hepatic Function Biomarker Assay

Serum alanine aminotransferase (ALT) and alkaline phosphatase (ALP) enzyme activities were assayed as described previously by Reitman and Frankel [22] and Tietz et al. [23]. Total protein (TP), albumin (Alb), and gamma glutamyl transferase (GGT) were estimated according to Gornall et al. [24], Westgard and Poquette [25], and Szasz [26], respectively. All the previously mentioned kits were purchased from DIACHEM Ltd. Co., Hungary.

#### 2.4.2. Lipid Profile Assay

Lipid profile calorimetric kits were purchased from Diamond diagnostic Co., Egypt. Serum total cholesterol (TC) was determined by enzymatic method as demonstrated by Allain et al. [27], and triglycerides (TGs) were performed according to Fossati and Prencipe [28]. High-density lipoprotein cholesterol (HDL-c) and low-density lipoprotein cholesterol (LDL-c) were determined as described by Bachorik [29].

### 2.5. Antioxidants and Oxidative Stress Measurements

#### 2.5.1. Preparation of Liver Homogenate

Liver tissues from different experimental groups were homogenized in ice cold 100 mM sodium phosphate-buffered saline (pH 7.4) and centrifuged at 5000 rpm for 30 min; thereafter, the obtained supernatants were kept at −80°C.

#### 2.5.2. Hepatic Antioxidants and Lipid Peroxidation Levels

Glutathione peroxidase (GPx), superoxide dismutase (SOD), and malondialdehyde (MDA) were assayed in liver homogenate according to Paglia and Valentine [30], Nishikimi et al. [31], and Mihara and Uchiyama [32], respectively. Previous kits were purchased from LifeSpan BioSciences Inc., USA, for GPx and OxisResearch, USA, for SOD and MDA.

#### 2.5.3. Cytochrome P450 Reductase (CYPR450)

Cytochrome P450 reductase was estimated by using ELISA kit (CUSABIO, China). The procedures were followed according to the manufacturer's protocol.

### 2.6. Histopathological Examination

Paraffinized liver tissue blocks were processed and cut on a microtome at 5 *μ*m thickness, then deparaffinized, and stained with hematoxylin and eosin (H&E) according to Drury and Wallington [33] in order to examine the hepatic histomorphological alterations.

### 2.7. Immunohistochemical (IHC) Evaluation of Caspase-3

Formalin-fixed paraffin-embedded specimens were cut into 4 *μ*m sections. After deparaffinization, sections were heated with an autoclave in Tris/HCl buffer (pH 9.0) for 20 min at room temperature for antigen retrieval. The sections were then incubated with 0.3% H_2_O_2_ in absolute methanol for 30 minutes and then incubated with primary antibody against caspase-3 (#PA1-29157, Thermo Fisher Scientific Co., USA) at concentration 1 : 1000. This was followed by sequential 60-minute incubations with secondary anti-rabbit antibody, Envision + System HRP-Labelled Polymer (Dako, USA), and visualization with liquid DAB (diaminobenzidine) substrate chromogen system (Dako, USA). All slides were lightly counterstained with hematoxylin for 30 seconds prior to dehydration and mounting [34].

### 2.8. Image Analysis of Caspase-3

Semi-quantitative method to assess the caspase-3 IHC staining intensity % from seven random fields/animal was proceeded using ImageJ program according to Elgawish et al. [35].

### 2.9. Liver Comet Assay

Single-cell suspensions were prepared from frozen livers according to the method described by Smith et al. [36]. The procedures for comet assay were followed as described by Abdelrazek et al. [37].

### 2.10. Statistical Analysis

Statistical analyses were made using SPSS software, v. 16.0 (SPSS Inc., IL, USA). All values were expressed as mean ± standard errors. One-way analysis of variance (ANOVA) followed by Duncan's multiple comparison tests was applied for analyzing values among groups. A probability level of *P* < 0.05 indicated significance.

## 3. Results

### 3.1. Body and Liver Weights

The rats in the BPA group showed numerically decreased F.B.wt than those in the control, although the decrease was not statistically significant. The decrease in body weight induced by BPA was slightly improved by LYC administration. Notwithstanding, the LYC-treated rats expressed significantly (*P* < 0.05) increased body weight in comparison with the BPA-treated rats. On the other hand, the B.wt.G of LYC rats was significantly (*P* < 0.05) increased in counter to the BPA rats which had significant reduction in B.wt.G when compared to the control rats. Moreover, BPA + LYC group was significantly (*P* < 0.05) improved than BPA group to a value comparable to control group. Concerning the absolute and relative liver weights, there were nonsignificant differences between groups (Table 1).

### 3.2. Serum Hepatic Function Biomarkers

Rats receiving BPA revealed significantly (*P* < 0.05) higher activities of serum ALT, ALP, and GGT enzymes and lower levels of TP and albumin than control rats. Meanwhile, BPA + LYC group exhibited significant (*P* < 0.05) improvement in these parameters when compared to the BPA group but still significantly (*P* < 0.05) differed from the control one. Rats supplemented with LYC only did not differ from the control group (Table 2).

### 3.3. Lipid Profile Assay

In the BPA-treated rats, the serum TC and LDL levels were significantly (*P* < 0.05) elevated in comparison with the control rats, whereas TC and LDL levels in rats receiving BPA + LYC were nonsignificantly altered compared to the control rats (Table 3).

### 3.4. Hepatic Antioxidative Status and Lipid Peroxidation

BPA significantly (*P* < 0.05) decreased GPx, SOD, and CYPR450 activities while increasing MDA level in comparison to control group. The SOD, GPx, and CYPR450 activities were significantly (*P* < 0.05) elevated while MDA level was significantly (*P* < 0.05) diminished in the BPA + LYC group in contrast to BPA group, thus improving the oxidative effect of BPA on liver tissue. In the LYC group, nonsignificant changes occurred compared to the control group (Table 4).

### 3.5. Histopathological Examination of the Liver

**Figure 1:**
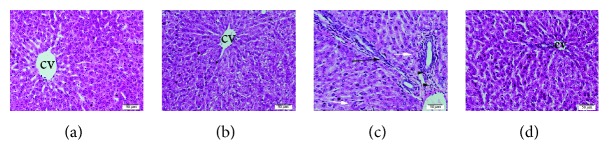
Histopathological sections of female Wistar rats. (a) Control group (b), LYC-treated (10 mg/kg) control, (c) BPA-treated (10 mg/kg) group, and (d) BPA (10 mg/kg) and LYC (10 mg/kg) cotreated group. (a) and (b) show normal hepatocytes arranged in radiating cords around central vein (cv). (c) The BPA-treated liver shows dilated vein, bridging fibrosis of portal areas (black arrow), mild leukocytic infiltration (arrowheads), and minute focal hepatocyte necrosis (white arrows). (d) BPA + LYC-treated liver shows amelioration of hepatic lesions with mildly vacuolization of hepatocytes.

The liver tissue of rats in the control and LYC groups showed normal hepatocytes with normal arrangement of hepatic cords around the central veins (Figures 1(a) and 1(b)). Livers of rats in BPA group exhibited fibrous expansion of some portal areas with occasional portal to portal bridging, foci of focal (spotty) lytic necrosis with dilated and congested central veins, and cytoplasmic vacuolization of hepatocytes with eccentric nuclei (Figure 1(c)), while rats in BPA + LYC group manifested no fibrous expansion of portal areas or necrosis with tendency for retaining normal hepatic architecture of hepatocytes accompanied with mild congestion of central vein (Figure 1(d)).

### 3.6. Immunohistochemical Evaluation (IHC) of the Liver

**Figure 2:**
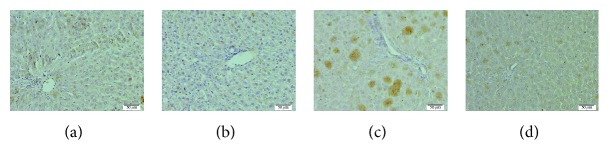
Immunohistochemical reaction of caspase-3 in livers of female Wistar rats. (a) Control group (b), LYC-treated (10 mg/kg) control, (c) BPA-treated (10 mg/kg) group, and (d) BPA (10 mg/kg) and LYC (10 mg/kg) cotreated group. Control and LYC groups show week immunoreactivity of caspase-3 while BPA-treated group exhibited higher immunoreactivity. The LYC coadministration with BPA shows amelioration of caspase-3 immunoreactivity than that in BPA alone.

The different experimental groups in this study showed variable positive staining intensities of caspase-3 using DAB chromogen, where BPA group rats revealed significant (*P* < 0.05) higher positive cytoplasmic staining area % of caspase-3 in the hepatocytes (Figure 2(c) and Table 4) than control (Figure 2(a) and Table 4), while BPA + LYC group showed significantly (*P* < 0.05) lower caspase-3 staining area % (Figure 2(d) and Table 4) than BPA group.

### 3.7. Liver Comet Assay

Comet tail DNA % showed a significant (*P* < 0.05) elevation in BPA-treated group than in control. Administration of LYC with BPA significantly (*P* < 0.05) reduced comet tail % than BPA group.

## 4. Discussion

BPA is a monomer found in plastic goods and affects adversely many organs especially the liver through induction of oxidation state. In this study, LYC was tested for its protective effect against the deleterious impacts induced by BPA in female rats' liver tissue. The present results demonstrated that BPA had no effect on body and liver weights. However, B.wt.G expressed significant decline in BPA-treated group than control while BPA + LYC showed improvement in B.wt.G to a level comparable to control. These results could be attributed to the toxic effect of BPA that could encounter several body homeostatic pathways, among which neuronal appetite loop in the brain [38], where BPA can easily cross blood brain barrier and have estrogenic effect [39]. Moreover, BPA can alter the antioxidant enzymes in different body systems [40, 41] as noted in the current study, thus could alter normal weight gain of the experimental rats. This can obviously illustrate the higher B.wt.G observed in BPA + LYC-treated group where LYC can alleviate oxidative stress [42] favor, cell-cell communication, and body performance in a positive manner [43, 44]. Our results were in harmony with Morrissey et al. [45] and Christiansen et al. [46] who recorded significant reduction in B.wt.G in BPA-treated female mice offspring and insignificant change in F.B.wt or organ weights of rats exposed to BPA, respectively. On the other hand, some reports in the literatures were inconsistent to our findings, where Rubin et al. [47] found significant increase in the body weight of Sprague Dawley female rats' offspring exposed to low BPA doses. The variation in results may be attributed to the diversity in diet composition, BPA exposure period, route and doses, and animal strain.

In our data, BPA expressed harmful effect on the liver evidenced by significant increase in the serum liver enzyme activities along with reduction in TP and albumin. The explanation of the higher hepatic enzyme activities could be due to altered hepatocytes' membrane permeability by BPA; thus, the cell membrane losses its functional integrity resulting in cellular leakage of these enzymes to circulation. This is augmented by the observed depletion in the activities of endogenous enzymatic antioxidants SOD, GPx, and CYPR450 that could increase hepatic membrane lipid peroxidation that disrupt its permeability [48]. Meanwhile, serum protein concentration is considered equilibrium between the rate of protein synthesis and breakdown. It is well known that BPA induces mitochondrial oxidative stress in the cell that results in protein damage [49], thus making protein damage more prominent than its synthesis. Moreover, administration of BPA disrupted hepatic integrity and functions where liver is considered the main organ involved in plasma protein biosynthesis [50]; thus, serum TP and albumin were declined. These obtained results were concurring with those recorded by Moon et al. [51], Geetharathan and Josthna [52], and Moustafa and Ahmed [53]. Fortunately, LYC reversed all abnormalities made by BPA intoxication and returned the values near normal. This finding harmonized with Sheriff and Devaki [54] and Jiang et al. [18] in other hepatic toxicity male rat models. Current results suggested that LYC consolidates hepatic cells' regeneration. Consequently, it strengthens the cellular membrane while diminishing the enzyme leakage and preserves its function in protein biosynthesis. The hepatic consolidating effect of LYC could be attributed to its ability for quenching of singlet oxygen and elimination of peroxyl radicals that was confirmed by the elevated antioxidant activities of SOD, GPx, and CYPR450 along with reduced MDA in BPA + LYC cotreated rats.

Administration of BPA in current study disrupted lipid metabolism that reflected negatively on serum profile results. Our results were in agreement with Moghaddam et al. [55] who recorded increased lipid profile in male mice treated with BPA for 4 weeks. BPA has the capability to disrupt the lipid metabolism [56] and trigger lipid accumulation through differentiation of 3T3-L1 fibroblasts into adipocytes [57]. The occurrence of abnormalities in lipid profile is considered the starting station for induction of oxidative stress and lipid peroxidation [58] that were observed in the current study. Administration of LYC with BPA, in this study, had improved the lipid profile. Jiang et al. [59, 18] confirmed our results as they suggested that LYC supplementation decreased TC, TGs, and LDL-c. LYC could decrease TC through diminishing cholesterol synthesis via inhibiting the hydroxy-methyl-glutaryl-coenzyme A reductase (HMGCoA), an enzyme controlling the rate of cholesterol formation besides declining LDL-c [60].

In our existing data, BPA evoked hepatic oxidative stress as it diminished activities of endogenous enzymatic antioxidant as SOD, GPx, and CYPR450 with increment in MDA which is an end product for lipid peroxidation. These results were in harmony with those obtained by Kabuto et al. [11], Asahi et al. [61], and Eid et al. [62]. The reduction in serum SOD activity could be due to excessive consumption in the autoxidation procedure induced by BPA in the liver. The decrease in SOD activity might lead to increase level of superoxide radicals which resulted in the inactivation of GPx [63], thus increasing hydrogen peroxide generation [11]. The observed increment in hepatic oxidative stress denoted by depletion of hepatic antioxidant enzymes could induce lipid peroxidation in hepatocytes' membrane, thus causing their damage [21, 64]. Moreover, the observed reduction in CYPR450, an essential enzyme for variable metabolic processes of xenobiotics and BPA metabolism [65], led to persistence of active BPA metabolites which further increased ROS production [66].

Otherwise, LYC offered a reciprocal impact on the liver tissue manifested by raised SOD, GPx, and CYPR450 activities along with lowered MDA level in rats treated with BPA + LYC. The antioxidant activity of LYC could be attributed to being a beta carotene, where LYC had been proved to protect against protein, lipid, and DNA oxidation [67] through scavenging singlet oxygen [68] and peroxyl radicals [69], thus limiting MDA production as lipid peroxidation end product [70].

Histopathological investigations augmented the previous results, where BPA resulted in deleterious hepatic changes ranged from hepatocytes' vacuolization with eccentric nuclei to focal necrosis and fibrosis. These results were similar to those obtained by Eid et al. [62]. Current histopathological picture was confirmative for the oxidative stress and lipid peroxidation induction nature of BPA observed in this study. Oxidative stress and lipid peroxidation led to destruction of hepatocytes' cell membrane and liberation of hepatic enzymes as well as perturbation in hepatic capacity for protein biosynthesis. The usage of LYC, as antioxidant, produced pronounced hepatic protection that markedly ameliorated the severity of hepatic lesions and subsequently the hepatic functions.

Hepatic homeostasis is gained through a regular cell turnover involving apoptosis of hepatocytes [71]. The increase of apoptosis via increment of DNA strand breaks with DNA migration from the nucleus into the comet tail together with increment in caspase-3 protein content in BPA group is an attribution for various types of the observed liver pathology. Current results were parallel to the previous results of Abdel Samie et al. [72] and Eid et al. [62]. Our data suggested that BPA increased caspase-3 apoptosis and DNA tail fragment breaks via depletion of antioxidant activities and lipid peroxidation. The observed hepatic oxidative stress due to BPA administration could possibly damage DNA. The administration of LYC with BPA significantly decreased DNA fragment % in comet tail as well as caspase-3 immunoreactive area % than BPA group. These results were in agreement with Kurcer et al. [73]. The possible attribution for LYC antiapoptotic effect is its antioxidant power as it is known for its free radical scavenging effect that reduces lipid peroxidation, protein, and DNA damage [74, 75].

## 5. Conclusion

In conclusion, the current study demonstrated the protective effects of LYC versus BPA hepatic oxidative injury and apoptotic effect by means of MDA suppression with SOD and GPx activities' amelioration. Antioxidant effects of LYC led to overregulation of CYPR450 that cleared BPA metabolites rapidly and decreasing the exposure of hepatic cells to their harmful effects. All these effects downregulated hepatic caspase-3, thus reducing apoptosis and thus keeping hepatic integrity, and prevented the liberation of hepatic enzymes into the blood of female Wistar rats.

## Figures and Tables

**Table 1:** Effect of LYC on body and liver weights of BPA-intoxicated female Wistar rats.

Parameter	Experimental groups			
	Control	LYC	BPA	BPA + LYC
F.B.wt (g)	164.80 ± 4.58^ab^	171.40 ± 9.60^a^	142.40 ± 3.53^b^	149.30 ± 11.81^ab^
B.wt.G (g)	54.20 ± 4.51^b^	70.40 ± 4.23^a^	34.40 ± 4.72^c^	53.90 ± 3.29^b^
Abs. liver wt. (g)	5.97 ± 0.43	6.56 ± 0.52	5.39 ± 0.19	5.40 ± 0.49
Rel. liver wt. (%)	3.61 ± 0.18	3.81 ± 0.10	3.79 ± 0.09	3.62 ± 0.16

Values are expressed as means ± SE (*n* = 7) in every group. BPA: bisphenol A; LYC: lycopene; F.B.wt: final body weight; B.wt.G: body weight gain; Abs. liver wt.: absolute liver weight; Rel. liver wt.: relative liver weight. Within the same row, means with different superscript letters differ significantly at *P* ≤ 0.05.

**Table 2:** Effect of LYC on serum biochemical hepatic markers in BPA-intoxicated female Wistar rats.

Parameter	Experimental groups			
	Control	LYC	BPA	BPA + LYC
ALT (U/l)	25.59 ± 0.10^c^	25.09 ± 0.11^c^	50.18 ± 1.05^a^	35.68 ± 1.07^b^
ALP (U/l)	66.93 ± 0.35^c^	66.20 ± 0.49^c^	93.19 ± 0.89^a^	74.51 ± 1.62^b^
GGT (U/l)	10.53 ± 0.12^c^	10.43 ± 0.11^c^	44.05 ± 0.79^a^	20.95 ± 0.55^b^
TP (g/dl)	6.10 ± 0.02^a^	6.14 ± 0.01^a^	4.94 ± 0.04^c^	5.54 ± 0.03^b^
Alb (g/dl)	4.60 ± 0.01^a^	4.61 ± 0.01^a^	3.64 ± 0.03^c^	4.05 ± 0.03^b^

Values are expressed as means ± SE (*n* = 7) in every group. BPA: bisphenol A; lycopene (LYC); ALT: alanine aminotransferase; ALP: alkaline phosphatase; GGT: gamma glutamyl transferase; TP: total protein; Alb: albumin. Within the same row, means with different superscript letters differ significantly at *P* ≤ 0.05.

**Table 3:** Effect of LYC on serum lipid profile in BPA-intoxicated female Wistar rats.

Parameter	Experimental groups			
	Control	LYC	BPA	BPA + LYC
TC (mg/dl)	102.00 ± 11.24^b^	101.67 ± 11.85^b^	146.33 ± 7.42^a^	119.67 ± 4.09^ab^
TGs (mg/dl)	81.00 ± 9.85^ab^	73.00 ± 9.87^b^	105.00 ± 2.52^a^	97.67 ± 6.94^ab^
HDL-c (mg/dl)	47.67 ± 3.93	52.00 ± 3.21	44.67 ± 3.71	47.33 ± 3.93
LDL-c (mg/dl)	52.53 ± 6.24^b^	40.87 ± 6.91^b^	83.13 ± 4.52^a^	49.80 ± 5.31^b^

Values are expressed as means ± SE (*n* = 7) in every group. BPA: bisphenol A; LYC: lycopene; TC: total cholesterol; TGs: triglycerides; HDL-c: high-density lipoprotein cholesterol; LDL-c: low-density lipoprotein cholesterol. Within the same row, means with different superscript letters differ significantly at *P* ≤ 0.05.

**Table 4:** Effect of LYC on hepatic tissue antioxidant enzyme activities, lipid peroxidation level, caspase-3 immunoreactivity, and comet tail DNA % in BPA-intoxicated female Wistar rats.

Parameter	Experimental groups			
	Control	LYC	BPA	BPA + LYC
GPx (nmol/mg)	99.06 ± 3.79^b^	108.85 ± 2.95^a^	61.14 ± 0.90^d^	88.62 ± 1.71^c^
SOD (U/mg)	7.60 ± 1.50^a^	7.62 ± 0.01^a^	5.74 ± 0.05^c^	6.74 ± 0.04^b^
MDA (nmol/mg)	0.54 ± 0.00^c^	0.52 ± 0.01^c^	0.93 ± 0.02^a^	0.72 ± 0.02^b^
CYPR450 (ng/g)	3.86 ± 0.04^a^	3.98 ± 0.01^a^	2.44 ± 0.04^c^	3.44 ± 0.08^b^
Caspase-3 IRA (%)	35.62 ± 4.82^c^	42.74 ± 5.71^c^	77.04 ± 2.97^a^	61.86 ± 3.09^b^
Comet tail DNA (%)	6.68 ± 1.04^c^	6.94 ± 1.29 ^c^	25.05 ± 2.93^a^	14.50 ± 2.61^b^

Values are expressed as means ± SE (*n* = 7) in every group. BPA: bisphenol A; LYC: lycopene, GPx: glutathione peroxidase; SOD: superoxide dismutase, MDA: malondialdehyde, CYPR450: cytochrome P450 reductase; IRA: immunoreactive area. Within the same row, means with different superscript letters differ significantly at *P* ≤ 0.05.
